# Cardiovascular risks and sociodemographic correlates of multidimensional sleep phenotypes in two samples of US adults

**DOI:** 10.1093/sleepadvances/zpac005

**Published:** 2022-02-18

**Authors:** Soomi Lee, Claire E Smith, Meredith L Wallace, Ross Andel, David M Almeida, Sanjay R Patel, Orfeu M Buxton

**Affiliations:** 1 University of South Florida, School of Aging Studies, Tampa, FL, USA; 2 University of Pittsburgh, Department of Psychiatry, Pittsburgh, PA, USA; 3 The Pennsylvania State University, Department of Human Development and Family Studies, State College, PA, USA; 4 University of Pittsburgh, Department of Medicine, Pittsburgh, PA, USA; 5 The Pennsylvania State University, Department of Biobehavioral Health, State College, PA, USA

**Keywords:** sleep health, sleep phenotypes, heart disease, cluster analysis, social disparities, middle adulthood

## Abstract

**Study Objectives:**

Sleep is a modifiable risk factor for cardiovascular conditions. Holistic examination of within-person, multidimensional sleep patterns may offer more detailed information about the sleep-cardiovascular condition link, including who is more vulnerable to both. This study aimed to identify common sleep phenotypes in adulthood, establish the validity of the phenotypes in relation to cardiovascular conditions, and explore sociodemographic and background characteristics of the phenotypes.

**Methods:**

Across two independent samples of adults (*N*_1_ = 4600; *N*_2_ = 2598) from the Midlife in the United States Study, latent class analysis (LCA) extracted sleep phenotypes using five key self-reported sleep dimensions. Log-binomial regression was used to determine whether sleep phenotypes differentially predicted cardiovascular conditions, adjusting for known risk factors. LCA with covariates was used to compare sociodemographic characteristics of the identified sleep phenotypes.

**Results:**

Four sleep phenotypes were identified consistently across the two samples: *good sleepers*, *nappers*, *dissatisfied/inefficient sleepers*, and *irregular sleepers*. Compared to *good sleepers* (reference), *dissatisfied/inefficient sleepers* exhibited a higher risk of cardiovascular conditions in both samples (*RR*_Sample1_: 29%, *RR*_Sample2_: 53%) and consisted of relatively more racial/ethnic minorities. *Nappers* exhibited a higher risk of cardiovascular conditions in one sample (*RR*_Sample1_: 38%) and consisted of more women and older adults. *Irregular sleepers* exhibited no significantly different cardiovascular risk and were relatively younger.

**Conclusions:**

Common sleep phenotypes in adulthood exhibit differential risks for cardiovascular conditions. Cooccurring sleep dissatisfaction and inefficiency, in particular, may relate to increased risk of cardiovascular conditions. Certain sociodemographic groups (racial minorities, women, older adults) disproportionately fit within high-risk sleep phenotypes.

**Statement of Significance**

In two samples of US adults, four common sleep phenotypes were identified: *good sleepers*, *dissatisfied/inefficient sleepers, nappers, and irregular sleepers.* Compared to *good sleepers*, *dissatisfied/inefficient sleepers* that included more racial/ethnic minorities, unmarried, and less educated people exhibited a higher risk of cardiovascular conditions consistently across the samples, after adjusting for sociodemographics and known risk factors. *Nappers* that included more older adults and women also exhibited a higher risk of cardiovascular conditions than *good sleepers* in one sample only. *Irregular sleepers* that included more younger adults did not exhibit a higher risk of cardiovascular conditions in either sample. These results provide an informed point of intervention jointly targeting co-occurring sleep problems in adulthood (e.g., dissatisfaction + inefficiency).

## Introduction

Poor sleep has serious physiological consequences that can culminate in cardiovascular disease [1, 2]. The connection between sleep and cardiovascular conditions is particularly pressing for certain vulnerable sociodemographic groups (e.g., racial/ethnic minorities, less educated, older adults) due to their heightened risk for both [3, 4]. As sleep is modifiable, improving sleep health may provide a low-cost opportunity to mitigate cardiovascular risk [5]. Buysse’s sleep health framework [6] emphasizes that thorough improvement of sleep health should not only target sleep duration but other critical sleep health dimensions, including regularity, satisfaction, daytime alertness, and efficiency. Indeed, one recent study measured people’s experiences of multiple sleep issues (i.e., as a composite score) to show that holistically optimal sleep across all measured dimensions related to 35% lower risk of cardiovascular disease compared with sleep that is *sub*optimal across all or all but one dimension [7]. Building on this initial evidence that multidimensional sleep health provides new and meaningful information about cardiovascular conditions, more detailed consideration of such patterns is needed [8] to expand beyond largely-good or largely-bad sleep to specific configurations of multiple sleep dimensions.

Previous research shows the importance of sleep in the risk of cardiovascular conditions, although a comprehensive picture on multidimensional sleep health is still lacking. For example, better sleep quality consistently relates to lower cardiovascular risk [9–11], but sleep duration and napping often exhibit curvilinear (i.e., U- or J-shaped) [12, 13] or inconsistent relations with cardiovascular outcomes [14, 15]. Sleep regularity and efficiency have been relatively understudied, though new evidence indicates their potentially significant roles in cardiovascular conditions [9, 16–18]. Yet sleep dimensions are not independent; multiple sleep characteristics may interactively predict cardiovascular conditions. In what Hall and colleagues [8] deem “one of the most important discoveries in sleep medicine in the past decade” (p. 5), short sleep duration predicts greater cardiovascular risk when *combined* with poor sleep quality but not when sleep quality is sufficient [19–21]. Joint effects between other, and more, sleep dimensions in predicting cardiovascular conditions have not yet been explored despite characterization of real-life sleep experiences as a constellation of multiple sleep dimensions [22], not just one or two dimensions in isolation.

Person-centered approaches provide the needed ability to clarify how various sleep experiences co-occur. Whereas typical variable-centered analyses such as regression assess relations between variables across people, person-centered analyses like latent class analysis (LCA) describe how variables relate to one another within people [23]. LCA identifies subgroups of people with similar standings on multiple variables [24]. In this way, LCA has the potential to describe people’s holistic patterns of various sleep dimensions, adding not only nuance but also realism to the measurement of sleep health as a pattern of co-occurring dimensions [25]. LCA can then be paired with variable-centered analyses to link identified classes to potential outcomes or covariates. Emerging research indicates that such analyses can indeed detect multidimensional sleep patterns that then enhance prediction of health outcomes such as diet and exercise [22], BMI [26], and all-cause and cardiovascular mortality [27]. Given the potential to identify complex and interactive patterns of sleep, person-centered approaches to sleep may be useful in enhancing prediction of cardiovascular conditions.

The present study examined the relationships between multidimensional sleep phenotypes and the risk of cardiovascular conditions. Because we aimed to describe sleep phenotypes common to the general adult population, we explored the validity and replicability of the empirically derived classes [24, 25] and their relative risks of cardiovascular conditions across two large, independent samples. Furthermore, we examined sociodemographic and background characteristics of the identified sleep phenotypes to understand who is more likely to have suboptimal sleep patterns and thus a higher risk of cardiovascular conditions.

## Methods

### Data

The present data were collected as part of the Midlife in the United States Study (MIDUS), which aims to understand the relationship between aging and health via a national probability sample. We used two separate samples in the present study: (1) MIDUS II (M2) and (2) MIDUS Refresher (MR). The M2 study was conducted in 2004–2009 as a follow-up to original MIDUS I survey and added an extensive sleep questionnaire [28]. The MR study was conducted in 2011–2014 to refresh and expand the MIDUS study by recruiting a new set of participants [29]. The two samples were independent; recruitment and data collection were conducted separately, and participation was mutually exclusive. The data used here include a one-time, self-report survey.

Originally, 5555 people participated in M2. Of the 4624 people who completed the M2 self-administered questionnaire (SAQ) that included sleep questions, two respondents were excluded due to insufficient responding on core sleep variables. Furthermore, respondents who provided extreme or non-plausible values on two sleep variables were excluded; 22 participants who reported >3 hours on sleep onset latency and 40 participants who reported >7-hour difference between weekdays and weekends (i.e., sleep irregularity) were excluded. The final M2 analytic sample consisted of 4600 people. Of the 3577 people in the MR sample, 967 did not complete the SAQ. Furthermore, 25 participants who reported >3 hours on sleep onset latency and 7 participants with >7-hour difference between weekdays and weekends were excluded. The final MR analytic sample consisted of 2598 people. The demographics of the analysis sample and those excluded from the analysis sample were compared in both M2 and MR (see Supplementary Table 1), but no large difference emerged (i.e., *d* <|.80| or *φ* <|.50|).

### Measures

#### Sleep characteristics.

Applying Buysse’s framework of sleep health, five fundamental sleep characteristics were assessed to represent a person’s sleep health: regularity, satisfaction, alertness, efficiency, and duration. The sixth characteristic in Buysse’s framework, timing, was not available in the MIDUS survey data and, therefore, could not be included. All sleep facets were dichotomized into undesirable (=1) or relatively desirable (=0) categories (Table 1) using *a priori* cutoffs derived from the sleep literature [30] and used as binary indicators in the LCA. *Regularity* was operationalized as the variation in sleep duration over the course of a typical week. Specifically, regularity was calculated via the absolute value of the difference between weekday and weekend sleep duration (irregular = |difference|>60 min vs. regular = |difference|≤60 min). *Satisfaction* was captured by four items concerning feeling unrested during the day, difficulty falling asleep, waking up during the night, and waking up too early in the morning, as is supported by previous research [31, 32]. If participants reported experiencing symptoms (sometimes, often, or almost always) in any of the four items, they were coded as dissatisfied (vs. satisfied). Based on Buysse’s guidance, *alertness* was operationalized as nap frequency [6] during a usual week (lack of alertness = # of naps>2 vs. # of naps≤2). Frequent naps (> 2 naps per week) have been found to be associated with incident cardiovascular events [33]. Also per Buysse’s guidance, *efficiency* was operationalized as sleep onset latency, or how long the respondent takes to fall asleep. While wake after sleep onset has been used to measure sleep efficiency based on actigraphy, sleep onset latency has been often used to capture sleep efficiency based on self-report [6] (inefficient = > 30 minutes vs. efficient = ≤ 30 minutes). *Duration* was operationalized as the typical amount of sleep the respondent gets on weekdays (suboptimal = ≤ 6 or ≥ 9 hours vs. optimal = 6 < hours < 9).

**Table 1.** Sleep facet measurement

Dimension	Domain	Assessment	Cut point
**R**egularity	Consistency of sleep duration	Difference between workday sleep duration and non-workday sleep duration	**Irregular:** absolute value > 60 minutes; **Regular:** absolute value ≤ 60 minutes
**S**atisfaction	Please indicate how often you experience each of the following:		**Dissatisfied:** sometimes, often, or almost always (on at least 1 of the 4 items) **Satisfied:** rarely or never (on all 4 items)
	Trouble falling asleep	Have trouble falling asleep.	
	Nocturnal awakenings	Wake up during the night and have difficulty going back to sleep.	
	Early awakenings	Wake up too early in the morning and be unable to get back to sleep.	
	Unrested upon waking	Feel unrested during the day, no matter how many hours of sleep you had.	
**A**lertness	Nap frequency	During a usual week, how many times do you nap for 5 minutes or more?	**Many naps**: > 2 **Few naps**: ≤ 2
**E**fficiency	Sleep latency	How long does it usually take you to fall asleep at bedtime?	**Inefficient**: > 30 minutes **Efficient**: ≤ 30 minutes
**D**uration	Workday sleep duration	How much sleep do you usually get at night (or in your main sleep period) on weekdays or workdays?	**Suboptimal duration**: ≤ 6 or ≥ 9 **Optimal duration**: > 6 & < 9

To conduct supplementary clustering analyses (i.e., LCA), the continuous sleep characteristics were dichotomized to reflect desirable and undesirable categories for each. These values were then used as indicators in LCA.

#### Prevalent cardiovascular conditions.

Cardiovascular conditions were measured using a binary indicator (0=no cardiovascular conditions, 1=one or more cardiovascular conditions). First, participants responded to the prompts, “Have you ever had heart trouble suspected or confirmed by a doctor?” and “Have you ever had severe pain across the front of your chest lasting half an hour or more?”. An affirmative response was followed up with: “What was the diagnoses – [condition type]?” for 10 conditions (yes or no response for each), namely (1) heart attack, (2) angina, (3) high blood pressure, (4) valve disease (including mitral valve prolapse, aortic insufficiency, bicuspid aortic valve), (5) hole in the heart (including atrial septal defect, ventricular septal defect), (6) blocked artery (including blocked/closed artery, coronary artery disease, coronary heart disease, and ischemia), (7) irregular heartbeat, (8) heart murmur, (9) heart failure, (10) other. High blood pressure was excluded because it is better characterized as a risk factor of cardiovascular disease rather than a core cardiovascular condition [34]. Furthermore, stroke was added as part of this binary indicator in core analyses, because it represents a common cardiovascular disease [35].

#### Covariates.

We considered sociodemographic characteristics that have been empirically associated with sleep and health, including age, sex, race/ethnicity (white and non-Latinx; non-white and/or Latinx), education (1–12 scale, from no/some grade school to doctorate), marital status (married and/or cohabitating; not married or cohabitating), and employment status (employed; unemployed; retired) [36–38]. Additionally, we included body mass index (BMI; in units of kg/m²), current smoking status (smoker; nonsmoker), and depression symptoms as covariates. Depression was assessed using six items from the World Mental Health Organization’s Composite International Diagnostic Interview Short Form [39]. To reduce overlap between this scale and sleep variables, an item measuring sleep problems (i.e., “have more trouble falling asleep than usual.”) was excluded from the scale. Participants indicated whether they experienced depression symptoms (e.g., “lose interest in most things”) for 2 weeks or more over the past 12 months (1=*yes*, 0=*no*), and a sum score of depression symptoms was created (*Range*=0 to 6).

#### Analytic strategy.

Using the five binary sleep characteristics as latent class indicators, we conducted LCA separately in the M2 and MR samples to determine the number and nature of sleep classes. Methodologists insist that such “replication is critical for person-centered research” (p. 808) [25]. Here, replication across the two independent samples can add rigor and test generalizability of the common sleep phenotypes within the general adult population. The LCAs were conducted in MPlus 7 using maximum likelihood. The appropriate class solution was holistically assessed using a variety of model fit statistics [40]. Specifically, Akaike Information Criterion (AIC), BIC, and Sample Size Adjusted BIC (SSA-BIC) compare fit between subsequent class solutions, with lower values indicating relatively better fit. Similarly, significant Bootstrap Likelihood Ratio Test (BLRT) and Vuong-Lo-Mendell-Rubin Likelihood ratio test (VLMR-LRT) indicate a comparatively better fit of each solution compared with the solution with one fewer class. Higher entropy, ranging from 0 to 1, suggests a more distinct classification of respondents into classes and, therefore, a more precise solution. Once the most appropriate solution was identified in each sample, respondents were sorted into one of the identified classes using the posterior probability (i.e., probability that a participant is assigned to a specific class based on their scores on the sleep characteristics). As such, LCA facilitates the creation of a categorical “class” variable, within which membership to each class is mutually exclusive.

Next, log-binomial regression in SAS 9.4 was used to examine whether sleep class membership significantly predicted the risk ratio of cardiovascular conditions, using the largest sleep class as a reference group and adjusting for both sociodemographics and known risk factors of cardiovascular conditions. Again, analyses were run separately for the M2 and MR samples. A risk ratio greater than one indicates the focal class relates to a higher risk of cardiovascular conditions than does the reference group; a risk ratio lower than one indicates the class relates to a lower risk of cardiovascular conditions than the reference group. A log-binomial strategy that outputs risk was chosen over a logistic regression strategy that outputs odds because odds ratios can overestimate risk of common outcomes (>10% prevalence) [41]. P-values were adjusted using the Benjamini-Hochberg method [42] for multiple comparisons.

Furthermore, to explore who was likely to belong to each sleep class, sociodemographic and background characteristics were included as covariates in LCA for the previously determined ideal class solution, separately for the M2 and MR samples. Again, the largest class was used as a reference group to assess whether the other class significantly differed from the reference group on these characteristics.

## Results

### Descriptive results

The M2 sample (*N* = 4600) was 55 years old on average, largely non-Hispanic white (79%), but relatively evenly distributed in terms of sex (56% female) and education (64% surpassed a high school degree). In terms of work status, 62% were working for pay, 25% were retired, and 13% were not employed. Similarly, the MR sample (*N* = 2598) was largely non-Hispanic white (82%) but relatively evenly distributed in terms of sex (53% female); they were more educated (78% surpassed a high school degree) and slightly younger (*M* = 52) than M2. In terms of their work status, 64% were working for pay, 24% were retired, and 12% were not employed. See Table 2 for more details on the two samples. Descriptive statistics and correlations among study variables are included in Table 3.

**Table 2.** Demographic information across the two samples

Category	*M* or % in M2	*M* or % in MR
**Sex**		
Male	43.70%	46.92%
Female	56.30%	53.08%
**Race**		
Non-Hispanic White	79.19%	81.59%
Black	17.02%	9.33%
Asian	0.54%	1.64%
All other races	3.24%	7.44%
**Education**		
Did not graduate high school	9.36%	5.97%
High school degree	26.64%	16.42%
Some college, no degree	21.62%	17.76%
College degree	25.12%	34.76%
> Bachelor’s degree	17.07%	25.09%
**Marital status**		
Unmarried	31.24%	30.49%
Married or cohabitating	68.76%	69.51%
**Work status**		
Worker	61.65%	64.00%
Retired	25.21%	23.62%
Not employed or retired (e.g., unemployed, laid off, disabled, homemaker, student, on leave)	13.14%	12.39%
**Work schedule**		
Nontraditional work schedule (i.e., works nights and/or weekends at least once per week)	20.33%	18.37%
Absence of nontraditional work schedule (i.e., nonworker or worker with a traditional work schedule)	79.67%	81.63%
**Smoking status**		
Current nonsmoker	83.70%	87.90%
Current smoker	16.30%	12.10%
**Body mass index (BMI)**	*M* = 28.32	*M* = 28.86
**Depression symptoms**	*M* = 0.54/6.00	*M* = 0.65/6.00

**Table 3.** Correlations and descriptive statistics

	M2		MR		1	2	3	4	5	6
	*M* or %	*SD*	*M* or %	*SD*						
1. Irregularity	0.65	1.01	0.69	0.95		.001	–.04*	.06**	–.23**	–.08**
2. Dissatisfaction	2.54	0.92	2.58	0.92	.001		.08**	.50**	–.33**	.10**
3. Nap frequency	2.09	2.56	1.86	2.46	–.03	.10**		.04*	–.08**	.07**
4. Inefficiency	0.44	0.45	0.47	0.45	.03	.49**	.01		–.19**	.08**
5. Duration	6.84	1.37	6.97	1.27	–.13**	–0.34**	–.10**	–.22**		.01
6. Cardiovascular conditions	19.37%		15.86%		–.08*	.12**	.13**	.07**	–.05**	

M2 correlations are below the diagonal. MR correlations are above the diagonal.

^**^ *p* < .001.

*p < .05.

### Identification of sleep phenotypes

LCA model fit statistics suggested a four-class solution in both M2 and MR samples (Supplementary Table 2). Although sample-size adjusted BIC (SSA-BIC) suggested a three-class solution, all other fit statistics suggested the four-class solution. Namely, the four-class solution exhibited the lowest AIC, highest entropy, and, most importantly, the last significant LMR and BLRT before the point of non-significance at the five-class solution.

**Figure 1.**
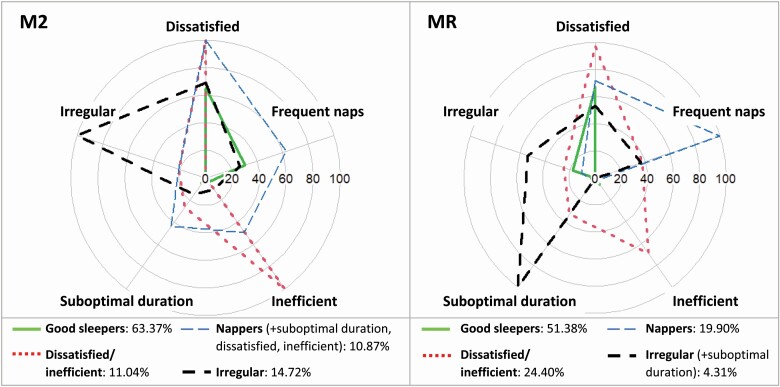
Percent of each sleep class belonging to the suboptimal sleep categories across M2 and MR. *Note*. These values represent the percentage of each sleep class belonging to the suboptimal sleep category (i.e., irregular, inefficient, dissatisfied, suboptimal duration, frequent naps). Sample-specific differences in sleep characteristics across the *napper* and *irregular sleeper* classes are noted in parentheses.

Next, the latent classes were named by their distinctive characteristics (Figure 1). The most common class (M2: 63.37%, MR: 51.38%) was characterized by optimal sleep duration and efficient sleep as well as regular sleep and infrequent napping; based on desirable standing across all sleep indicators, this class was labeled *good sleepers*. The second class (M2: 11.04%, MR: 24.40%) was characterized by dissatisfaction and inefficiency and thus was labeled *dissatisfied/inefficient sleepers*. The third class (M2: 10.87%, MR: 19.90%) was primarily characterized by frequent naps and was, therefore, labeled *nappers*. Of note, the *napper* group exhibited otherwise desirable sleep characteristics in MR (i.e., optimal duration, efficient sleep) but undesirable sleep characteristics in M2 (i.e., suboptimal duration, dissatisfaction, inefficiency). Finally, the fourth class (M2: 14.72%, MR: 4.31%) was characterized by irregularity in both M2 and MR and was, therefore, labeled *irregular sleepers*. However, a key difference among *irregular sleepers* between M2 and MR was that *irregular sleepers* in MR were characterized by suboptimal sleep duration, while irregular sleepers in M2 appeared desirable on all other sleep characteristics in MR. Overall, *good sleepers* and *short/dissatisfied sleepers* were fully replicated across both samples; *irregular sleepers* and *nappers* were partially replicated in that each shared one defining feature across samples but also demonstrated at least one sample-specific difference across other sleep dimensions. Such differences are common and do not prohibit subsequent steps connecting classes to additional variables [43].

### Associations between the identified sleep phenotypes and cardiovascular conditions

**Figure 2.**
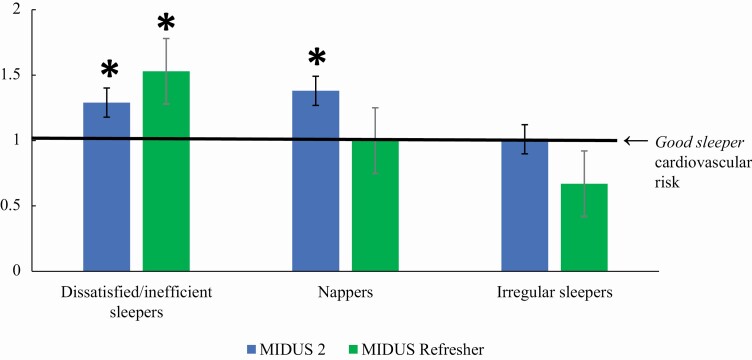
Risk ratios of cardiovascular conditions by sleep class, adjusted for covariates. *Note.* Good sleepers are the reference group. * indicates that the risk of cardiovascular conditions is significantly different from that of good sleepers (i.e., significantly different from 1 as marked by the solid black line).

Table 4 and Figure 2 report the risk of cardiovascular conditions predicted by the identified sleep classes. After adjusting for all covariates, compared with *good sleepers* (reference group)*, dissatisfied/inefficient sleepers* exhibited 29% higher risk of cardiovascular conditions in M2 and 53% higher risk of cardiovascular conditions in MR. *Nappers* exhibited a 38% higher risk of cardiovascular conditions than *good sleepers* in M2 but no significantly higher risk in MR. Finally, *irregular sleepers* did not exhibit significantly different cardiovascular risks than *good sleepers* in either sample. Significance did not change following Benjamini-Hochberg adjustment for multiple tests. Also of note, results were similar when stroke was excluded from the indicator of cardiovascular conditions (Supplementary Table 3).

**Table 4.** Log-binomial regression of sleep classes predicting cardiovascular conditions in M2 and MR.

		Unadjusted				Adjusted			
M2 Sample	**Sleep class**	**β [CI]**	**S.E.**	**Risk Ratio [CI]**	**S.E.**	**β [CI]**	**S.E.**	**Risk Ratio [CI]**	**S.E.**
	Dissatisfied/ inefficient	.22^*+^ [.05,.38]	.08	1.25^*+^ [1.05, 1.47]	.11	.25^*+^ [.09,.42]	.09	1.29^*+^ [1.09, 1.52]	.11
	Nappers (+poor night sleep)	.53^*+^ [.39,.67]	.07	1.71^*+^ [1.48, 1.96]	.12	.32^*+^ [.18,.46]	.07	1.38^*+^ [1.20, 1.58]	.10
	Irregular (+good night sleep)	–0.30^*+^ [–.49, –.10]	.10	0.74^*+^ [0.61, 0.90]	.07	.01 [–.19,.20]	.10	1.01 [0.83, 1.58]	.10
MR Sample	**Sleep class**	**β [CI]**	**S.E.**	**Risk Ratio [CI]**	**S.E.**	**β [CI]**	**S.E.**	**Risk Ratio [CI]**	**S.E.**
	Dissatisfied/ inefficient	.56^*+^ [.37,.75]	.10	1.75^*+^ [1.44, 2.11]	.17	.42^*+^ [.13,.71]	.15	1.53^*+^ [1.14, 2.04]	.23
	Nappers (+good night sleep)	.37^*+^ [.16,.59]	.11	1.45^*+^ [1.17, 1.80]	.16	.004 [–.30,.31]	.15	1.00 [0.74, 1.36]	.15
	Irregular (+suboptimal duration)	–.19 [–.72,.33]	.27	0.82 [0.49, 1.40]	.22	–.39 [–1.090,.30]	.35	0.67 [0.33, 1.35]	.24

^+^ indicates a statistically significant *p*-value (<.05) following Benjamini-Hochberg adjustment for multiple tests. * indicates statistical significance based on raw CI. CIs not containing 0 indicate a significant coefficient. CI not containing 1 indicates a significant risk ratio. Smoking status, BMI, depression, age, sex, race/ethnicity, education, marital status, and work status were included as covariates. Good sleepers were used as the reference group. Standardized coefficients are presented.

### Associations between sociodemographic and background characteristics and the identified sleep phenotypes

Table 5 reports the associations between sleep classes and both sociodemographic and background factors, relative to the *good sleeper* class in M2 and MR, separately. To test these associations, the LCAs were run using three separate models: (1) with sociodemographic characteristics as covariates, (2) with work status variables as covariates, and (3) with risk factors for cardiovascular conditions as covariates. The sleep classes exhibited the same defining characteristics in these results; thus, we were able to compare their sociodemographic and background characteristics. In both M2 and MR samples, non-White, unmarried, and less educated were more likely to belong to *dissatisfied/inefficient sleepers* relative to *good sleepers*. Older adults and women were more likely to belong to *nappers* than *good* sleepers in both samples; in MR, people not living with partners (marital, cohabitation) and with less education were also relatively more likely to belong to *nappers*. Younger people were more likely to belong to *irregular sleepers* than *good sleepers* in both samples. In M2, workers were also relatively more likely to belong to *irregular sleepers*; unpartnered and less educated people were also relatively more likely to belong to *irregular sleepers* in MR.

**Table 5.** Covariates of the sleep classes in M2 and MR using good sleepers as the reference group

Sample	Sleep class	Age	Sex(0 = female, 1 = male)	Race/Ethnicity (0 = minority, 1 = non-Hispanic white)	Marital status (0 = not partnered, 1 = married/ cohabitating)	Education	Worker (0 = retired or unemployed, 1 = worker)	Retired (0 = worker or unemployed, 1 = retired)	Unemployed (0 = worker or retired, 1 = unemployed)	Smoker status (0 = nonsmoker, 1 = smoker)	BMI	Depression
**M2**	*Dissatisfied/ inefficient*	0.01 (*p* = .78)	–0.04 (*p* = .91)	**–1.03 (*p* < .001)**	**–0.76 (*p* = .002)**	**–0.21 (*p* < .001)**	–0.54 (*p* = .66)	0.85 (*p* = .15)	0.91 (*p* = .18)	**0.28 (*p* = .04)**	**.04 (*p* < .001)**	**.47 (*p* < .001)**
	*Nappers*	**0.08 (** *p* **= .005)**	**–1.13 (** *p* **< .001)**	–0.42 (*p* = .15)	–0.37 (*p* = .24)	–0.06 (*p* = .14)	–0.43 (*p* = .74)	0.73 (*p* = .14)	0.16 (*p* = .87)	**0.62 (*p* < .001)**	**0.07 (*p* < .001)**	**0.64 (*p* < .001)**
	*Irregular sleepers*	**–.02 (*p* = .03)**	–0.13 (*p* = .58)	–0.74 (*p* = .29)	–0.25 (*p* = .39)	–0.14 (*p* = .22)	**83.92 (*p* < .001)**	–0.88 (*p* = .61)	**–85.85 (*p* < .001)**	–0.07 (*p* = .53)	0.01 (*p* = .52)	**0.24 (*p* < .001)**
**MR**	*Dissatisfied/ inefficient*	.003 (*p* = .85)	–.05 (*p* = .90)	**–.98 (*p* < .001)**	**–1.19 (*p* < .001)**	**–.39 (*p* < .001)**	–1.08 (*p* = .69)	.44 (*p* = .69)	.17 (*p* = .89)	**.97 (*p* = .001)**	**.08 (*p* < .001)**	**.53 (*p* < .001)**
	*Nappers*	**.09 (*p* < .001)**	**–1.51 (*p* = .001)**	.22 (*p* = .70)	**–.81 (*p* = .05)**	**–.19 (*p* = .005)**	–3.53 (*p* = .35)	–2.49 (*p* = .49)	–2.96 (*p* = .45)	.29 (*p* = .16)	.**06 (*p* < .001)**	**.50 (*p* < .001)**
	*Irregular sleepers*	**–.02 (*p* = .01)**	–.19 (*p* = .24)	–.22 (*p* = .22)	**–.71 (*p* < .001)**	**–.09 (*p* = .004)**	–.68 (*p* = .78)	–2.57 (*p* = .28)	–1.92 (*p* = .43)	.55 (*p* = .13)	.04 (*p* = .07)	.03 (*p* = .86)

Note. Unstandardized coefficients. Three models were run: one including all sociodemographic covariates, one including work status covariates, and one including risk factor covariates. Significant relations are in bold.

In terms of background health characteristics, those with higher BMI and depression were more likely to belong to *dissatisfied/inefficient sleepers* or *nappers* than *good sleepers* in both samples. Current smokers were more likely to be *dissatisfied/inefficient sleepers* than *good sleepers* in both samples and *nappers* than *good sleepers* in M2 only. Finally, those with higher depression were more likely to belong to *irregular sleepers* than *good sleepers* in M2, but no measured background health characteristics were significantly associated with the probability of belonging to *irregular sleepers* relative to *good sleepers* in MR.

### Supplementary analyses

As a further test of the value of multidimensional, within-person sleep phenotypes when predicting cardiovascular conditions, we compared their prediction to that by the individual (binary) sleep characteristics on their own. We first compared the effect sizes of these predictions to determine whether one approach provided substantially more information than the other. Some statistical considerations should be noted when interpreting these findings. Person-centered approaches like the LCA model complex interactions between multiple variables as they exist within people [44]. Due to downward biasing resulting from statistical and measurement artifacts, even simple interactions (e.g., one-way moderations with only two variables interacting) tend to exhibit small effect sizes compared with main effects [45]. Here, sleep classes model interactions between the *five* sleep dimensions in predicting cardiovascular conditions. Thus, we expected the individual sleep characteristics to significantly predict cardiovascular conditions—and potentially to an even greater extent than did the more complex sleep classes. As expected, the individual categorical sleep characteristics did significantly associate with cardiovascular conditions after adjustment for covariates in many cases; all dimensions except irregularity were significant predictors in M2, and dissatisfaction and inefficiency were significant in MR (Supplementary Table 4). That said, despite the increased statistical challenge of detecting significant effects when using sleep classes, the risk ratios of cardiovascular conditions were comparable in magnitude regardless of whether sleep was modeled as independent dimensions or multidimensional classes. The significant risk ratios of cardiovascular conditions based on sleep characteristics ranged from 1.27 to 1.48 (Supplementary Table 4), and the adjusted risk ratios provided by the sleep classes (Table 4) ranged from 1.29 to 1.53.

We next tested the covariate-adjusted associations of the five individual binary sleep characteristics with cardiovascular conditions (step 1) followed by the incremental prediction of cardiovascular conditions by the sleep classes above and beyond each individual sleep characteristic (step 2). Of note, we assessed multicollinearity between the predictors given expected interrelations between sleep dimensions and phenotypes; however, all variance inflation factors (VIF) were between 1 and 3, suggesting moderate correlations but not extensive multicollinearity that would threaten the validity of our results [46]. Supplementary Table 5 depicts these results in detail. Most incremental validity tests (step 2) were significant, with exceptions being that the *dissatisfied/inefficient* phenotype only reached marginal significance (i.e.,.05 < *p* < .10) over most sleep characteristics in the M2 sample and the *irregular sleeper* phenotype did not significantly predict cardiovascular conditions above any individual sleep characteristics in the MR sample. Altogether, these results showed that both individual sleep dimensions and the four sleep classes were significantly associated with cardiovascular risk—and to comparable magnitudes—but that the sleep classes still provided additional information (i.e., incremental prediction) beyond the sleep dimensions alone in many cases.

## Discussion

The present study provides the relative cardiovascular risk of four mutually exclusive multidimensional sleep phenotypes, which capture within-person configurations of five key sleep characteristics [6]. *Dissatisfied/inefficient sleepers*, in both samples, and *nappers*, in one of the two samples, were at greater risk of cardiovascular conditions than *good sleepers*. Moreover, the associations found between the sleep phenotypes and sociodemographic characteristics suggest who may be more likely to belong to these at-risk sleep phenotypes. Namely, racial and ethnic minorities were more likely to belong to *dissatisfied/inefficient sleepers* whereas older people and women were more likely to belong to *nappers*. Our findings further point to the co-occurrence of sleep dissatisfaction and inefficiency as a risk factor for cardiovascular conditions and specify the sociodemographic characteristics of those likely to exhibit these sleep patterns.

### Relative risk of cardiovascular conditions across four sleep phenotypes

The present results indicate that multidimensional sleep phenotypes provide rich and unique information about cardiovascular risk. Across two samples, we identified four latent sleep classes: *good sleepers*, *dissatisfied/inefficient sleepers*, *nappers*, and *irregular sleepers*. *Good sleepers* and *dissatisfied/inefficient sleepers* were highly similar across both samples and thus demonstrated, especially strong construct validity and generalizability. The remaining two classes shared key defining features across both samples (i.e., frequent napping for the *napper* class and irregular sleep duration throughout the week for the *irregular sleeper* class) but also displayed some sample-specific differences, which we consider in our discussion below. All of this acknowledged, the four resulting sleep phenotypes differentially related to cardiovascular risk in the two independent samples, above and beyond sociodemographics and other established risk factors (i.e., smoking, BMI, and depression). In general, *good sleepers* and *irregular sleepers* exhibited comparatively low cardiovascular risk, whereas *dissatisfied/inefficient sleepers* and *nappers* (in M2 sample only) exhibited relatively higher cardiovascular risk. Joint consideration of multiple sleep dimensions as they occur within people seems to provide additional predictive information about this critical health outcome. These results offer several novel contributions.

First, the multidimensional sleep phenotypes advance findings from emerging sleep composite score research, which suggests that more co-occurring sleep problems relate to a higher risk of cardiovascular conditions [7]. In line with this finding, in our study, *good sleepers* experienced optimal sleep characteristics across multiple dimensions and exhibited a relatively low risk of cardiovascular conditions compared with suboptimal sleep phenotypes. However, further adding to these previous findings, *irregular sleepers* exhibited no significantly higher cardiovascular risk than *good sleepers* despite experiencing one suboptimal sleep characteristic (irregular sleep duration across the week) in the M2 sample and two suboptimal sleep characteristics (irregular duration and suboptimal weekday sleep duration) in the MR sample. Although early evidence has previously suggested that irregular sleep, on its own, may be a risk factor for cardiovascular conditions [16], irregular sleep—both as an individual dimension and in the context of otherwise good sleep as seen in *irregular sleepers—*did not pose a significant risk in either of our samples. Additional research on irregular sleep is needed given these inconsistent findings. Still, the comparable cardiovascular risk of *good sleepers* (with no sleep issues) and *irregular sleepers* (with one to two sleep issues) is evidence that holistic sleep patterns may provide nuance to the prediction of cardiovascular conditions beyond previous approaches simply measuring the number of sleep issues a person experiences.

Relatedly, our findings indicate that frequent daytime napping may only pose a significant risk of cardiovascular conditions in the context of otherwise poor nighttime sleep. Whereas *nappers* in the MR sample had otherwise good sleep and no higher cardiovascular risk than *good sleepers*, M2 *nappers* simultaneously experienced dissatisfying, inefficient, and suboptimal-duration sleep and had 38% higher cardiovascular risk. Although occasional napping has been previously found to compensate for habitually insufficient duration or dissatisfaction during nighttime sleep when predicting cardiovascular events [33], our results demonstrate that the co-occurrence of frequent napping with habitually poor nighttime sleep is a risk factor for cardiovascular conditions rather than a protective factor. This finding could be explained by previous research showing that frequent naps may be inadequate to counteract the negative consequences of chronically poor nighttime sleep [47], but future research should explicitly test this explanation.

Finally, the cooccurrence of dissatisfying and inefficient sleep was identified as a consistent risk factor for cardiovascular conditions across the two samples. *Dissatisfied/inefficient sleepers* were at 29% higher risk of cardiovascular conditions than *good sleepers* in the M2 sample and 53% higher risk in the MR sample. Further underlining the seemingly critical joint role of these two characteristics, cooccurring dissatisfying and inefficient sleep (along with frequent napping and suboptimal duration) were again associated with heightened cardiovascular risk (38%) in M2 *nappers*. These two sleep characteristics may indicate insomnia symptoms, which involve difficulty falling or staying asleep [48]. Insomnia is generally associated with greater cardiovascular risk [49, 50], but inconsistent definition and measurement of insomnia [51] obscure the combination of sleep problems central to this risk. We expected sleep dissatisfaction to emerge as a consistent component of high-risk sleep phenotypes based on past findings that it is a key predictor of cardiovascular conditions and, importantly, that satisfying sleep can even compensate for a suboptimal duration to protect against cardiovascular conditions [52]. Sleep inefficiency was previously identified as another important, but relatively understudied, predictor of cardiovascular conditions on its own [17, 18]. In total, our findings on the heightened cardiovascular risk of coexisting sleep dissatisfaction and inefficiency answer calls to “dissect the distinct as well as overlapping influences” (p. 442) of various sleep issues, especially those relevant to insomnia, in predicting cardiovascular risks [53]. Future research should examine the potentially distinct pathophysiological mechanisms [53] that connect the *dissatisfied/inefficient* and *napper (+suboptimal nighttime sleep)* phenotypes to heightened cardiovascular risk.

### Sociodemographic and background characteristics of the sleep phenotypes

Our results also indicate which groups may be at greater risk of cardiovascular conditions due to their sleep experiences. The most consistent high-risk phenotype, *dissatisfied/inefficient sleepers,* had a greater proportion of racial and ethnic minorities, unmarried, and less educated people compared with *good sleepers*. While social disparities in sleep are well known [54], our findings suggest that these groups may benefit from interventions that jointly address sleep satisfaction and efficiency, such as a combined physical activity and sleep intervention [55], to potentially reduce their cardiovascular risk. Next, *nappers*, consisting of more women and older adults, were also a high-risk phenotype in the M2 sample. This finding aligns with previous research showing that older adults experience more daytime sleepiness and schedule flexibility that facilitate napping [56] and that, in turn, napping relates to higher cardiovascular risk for older adults specifically [57]. Conversely, sex differences in napping prevalence are less consistent in the literature [58, 59], but there is emerging evidence that women’s napping is more strongly associated with heightened cardiovascular risk than is men’s [57, 60]. Our results suggest that efforts toward reducing cardiovascular risk in older adults and women should focus on napping and suboptimal nighttime sleep. Based on past work, targeted sleep interventions for these groups could include mindfulness meditation, since it can jointly improve nighttime sleep and daytime function [61].

As a final consideration, *irregular sleepers* consisted of relatively younger adults compared with *good sleepers*, potentially driven by the irregularity in overall lifestyle and schedule seen in younger ages [62]. The younger age that characterizes the *irregular sleeper* phenotype in the present study may provide a more coherent explanation for why this group did not experience greater cardiovascular risk here despite previous findings to the contrary in variable-centered research using an older sample [16]. Namely, age itself is a risk factor for cardiovascular conditions because exposure to other cardiovascular risk factors naturally increases as a person ages [63]; it seems plausible that, along this same vein, the heart-damaging effects of sleep irregularity may take time to accumulate over the lifespan and thus emerge more consistently in older adults. More work is needed to understand the complex relations between age, sleep irregularity, and cardiovascular conditions. However, the *irregular sleeper* phenotype identified in the current study appears to be younger and at relatively low risk of cardiovascular conditions.

### Limitations and future directions

Despite the new information this study provides about the relations between co-occurring sleep health problems, cardiovascular conditions, and sociodemographic and background characteristics, certain limitations should also be considered. First, all data were collected at one time point via self-report surveys. This design could result in inaccurate measurement due to biases related to memory [64] or social desirability [65]. Future research should attempt to replicate the sleep phenotypes identified here using objectively measured sleep characteristics (e.g., using actigraphy [66]) and their longitudinal associations with a wider variety of health outcomes including mortality. Further replication attempts may also answer questions raised by the partial replicability of the *napper* and *irregular sleeper* phenotypes identified here, to determine whether more consistent characteristics are observed across additional samples or if drivers of variation (e.g., sample age, race/ethnicity) can be determined. In these future efforts, researchers could also go beyond dichotomization of sleep characteristics into optimal and suboptimal groups to provide more nuanced description (e.g., short, sufficient, and long sleep [27]). Other operationalizations of the sleep dimensions (e.g., daytime alertness as subjective sleepiness ratings during waking hours; efficiency as wake after sleep onset) and inclusion of other important dimensions (e.g., timing) should be considered in future research to measure sleep health more comprehensively [6] than was possible in the present study given the use of secondary data.

It may also be informative to use stratified analyses to determine whether these relationships manifest differently across vulnerable sociodemographic groups (i.e., minorities, women, older adults). In addition, we would encourage future researchers to study the mechanisms connecting sleep phenotypes to cardiovascular conditions to increase potential for effective and informed interventions. Finally, in this study, we used physician-diagnosed cardiovascular conditions as the outcome, which includes diverse etiologies and some congenital conditions (e.g., hole in heart and valvular disease). Future studies could examine cardiovascular disease, with a focus on atherosclerotic diseases as a potential consequence of poor sleep in midlife [67, 68], to see how the present findings extend to related cardiovascular risks and clarify specific disease processes involved.

## Conclusion

Sleep is a key and modifiable risk factor for cardiovascular conditions [54] and should, therefore, be studied with great detail, complexity, and realism. Using LCA, the present study demonstrates that multidimensional sleep phenotypes predict cardiovascular risk above and beyond other critical risk factors. Moreover, our findings suggest that the specific combinations of sleep health problems present within a person offer new information beyond that offered by each individual sleep characteristic. The most consistent risk factor for cardiovascular conditions identified in the present study was the co-occurrence of sleep dissatisfaction and inefficiency. People who are unmarried, less educated, and/or racial or ethnic minorities may be more vulnerable to this *dissatisfied/inefficient sleeper* phenotype, potentially explaining their heightened cardiovascular risk in previous research and providing an informed point of intervention jointly targeting these two sleep characteristics.

## Supplementary Material

zpac005_suppl_Supplementary_Tables
