# Supplementary material for: Cardiovascular risks and sociodemographic correlates of multidimensional sleep phenotypes in two samples of US adults
Source: Sleep Adv. 2022 Feb 18;3(1):zpac005. doi: 10.1093/sleepadvances/zpac005 (PMC8918427; doi:10.1093/sleepadvances/zpac005)
Supplement: zpac005_suppl_Supplementary_Tables [file zpac005_suppl_supplementary_tables.docx]

Cardiovascular Risks and Sociodemographic Correlates of Multidimensional Sleep Classes in Two Samples of U.S. Adults

Soomi Lee, PhD^*,1^, Claire E. Smith^1^, Meredith L. Wallace^2^, Ross Andel^1^, David M. Almeida^3^, Sanjay R. Patel^4^, Orfeu M. Buxton^5^

^1^University of South Florida, School of Aging Studies, Tampa, FL, U.S.A; ^2^University of Pittsburgh, Department of Psychiatry, Pittsburgh, PA, U.S.A.; ^3^The Pennsylvania State University, Department of Human Development and Family Studies, State College, PA, U.S.A.; ^4^University of Pittsburgh, Department of Medicine, Pittsburgh, PA, U.S.A.; ^5^The Pennsylvania State University, Department of Biobehavioral Health, State College, PA, U.S.A.

*** Address correspondence to**:

Soomi Lee, PhD.

Assistant Professor, School of Aging Studies

University of South Florida

4202 E. Fowler Avenue, MHC 1344,

Tampa, FL 33620

Email: [soomilee@usf.edu](mailto:soomilee@usf.edu)

**Supplemental Table 1. Analysis and excluded sample differences across M2 and MR.**

|  | **M2** | | | **MR** | | |
| --- | --- | --- | --- | --- | --- | --- |
|  | *X^2^* or *t* test | Effect size | Difference | *X^2^* or *t* test | Effect size | Difference |
| Age | *t*(5552)=8.41*** | *d*=.30 | Analysis sample (*M*=56)**>**excluded group (*M*=52) | *t*(3574)=11.51*** | *d*=.43 | Analysis sample (*M*=52)**>**excluded group (*M*=46) |
| Sex | *X^2^*(1,5554)=44.12*** | *φ*=.09 | Analysis sample (56% women)>excluded group (44%) | *X^2^*(1,4076)=491.69*** | *φ*=.34 | Analysis sample (53% women)>excluded group (49%) |
| Race | *X^2^*(1,5549)=4.49*** | *φ*=.03 | Analysis sample (21% minority members)**>**excluded group (18%) | *X^2^*(1,3575)=5.28* | φ=.04 | Analysis sample (18% minority members)**>**excluded group (8%) |
| Education | *t*(5545)=0.84 | *d*=.03 | n.s. | *t*(3567)=5.01*** | *d*=.19 | Analysis sample (*M*=7.92/12)**>**excluded group (*M*=7.45/12) |

*Note. *=statistical significance (p<.05). In M2, N(analysis sample)=4600 and N(excluded group)=955. In MR, N(analysis sample)=2598 and N(excluded group)=1486.*

**Supplemental Table 2.** Model fit statistics for M2 and MR samples.

| **Sample** | **No. of profiles** | **Free parameters** | **LL** | **AIC** | **SSA-BIC** | **Entropy** | **Adjusted LMR(*p*)** | **BLRT(*p*)** |
| --- | --- | --- | --- | --- | --- | --- | --- | --- |
| M2 | 1 | 5 | -11947.53 | 23905.07 | 23921.35 |  |  |  |
|  | 2 | 11 | -11673.83 | 23369.83 | 23405.65 | .48 | 536.63 (p<.001) | -11947.53 (p<.001) |
|  | 3 | 17 | -11654.57 | 23343.14 | **23398.50** | **.67** | 37.94 (p<.001) | -11673.92 (p<.001) |
|  | 4 | 23 | -11639.03 | **23324.05** | 23398.95 | **.75** | **30.49 (p<.001)** | **-11654.57 (p<.001)** |
|  | 5 | 29 | -11636.29 | 23330.58 | 23425.01 | **.62** | 5.37 (p=.28) | -11639.03 (p=1.00) |
| MR | 1 | 5 | -6738.99 | 13487.97 | 13501.40 |  |  |  |
|  | 2 | 11 | -6597.42 | 13216.83 | 13246.37 | .59 | 277.26 (p<.001) | -6738.99 (p<.001) |
|  | 3 | 17 | -6564.18 | 13162.35 | **13208.00** | **.73** | 65.10 (p<.001) | -6597.42 (p<.001) |
|  | 4 | 23 | -6554.59 | **13155.17** | 13216.93 | **.78** | **18.78 (p<.001)** | **-6564.18 (p<.001)** |
|  | 5 | 29 | -6552.67 | 13163.33 | 13241.20 | **.61** | 3.76 (p=.80) | -6554.59 (p=1.00) |

*Note.* The best values (in terms of model fit) are bolded. Grey highlighted cells indicate the selected profile solution.

**Supplemental Table 3.** Log-binomial regression of sleep classes predicting cardiovascular conditions (excluding stroke) in M2 and MR.

|  |  | **Unadjusted** | | **Adjusted** | |
| --- | --- | --- | --- | --- | --- |
| M2 Sample | **Sleep class** | **β [CI]** | **Risk Ratio [CI]** | **β [CI]** | **Risk Ratio [CI]** |
|  | Dissatisfied/ inefficient | .16 [-.02, .34] | 1.17 [.98, 1.41] | .21* [.03, .40] | 1.24* [1.03, 1.49] |
|  | Nappers (+poor night sleep) | .55* [.40, .70] | 1.73* [1.49, 2.01] | .35* [.20, .49] | 1.41* [1.22, 1.64] |
|  | Irregular (+good night sleep) | -.26* [-.46, -.06] | .77* [.63, .94] | .05 [-.16, .25] | 1.05 [.86, 1.28] |
| MR Sample | **Sleep class** | **β [CI]** | **Risk Ratio [CI]** | **β [CI]** | **Risk Ratio [CI]** |
|  | Dissatisfied/ inefficient | .53* [.32, .73] | 1.69* [1.38, 2.07] | .37* [.07, .67] | 1.44* [1.07, 1.95] |
|  | Nappers (+good night sleep) | .39* [.17, .62] | 1.48* [1.18, 1.85] | .01 [-0.30, .32] | 1.00 [.74, 1.37] |
|  | Irregular (+suboptimal duration) | -.20 [-.75, .35] | .82 [.47, 1.43] | -.32 [-1.01, .37] | .73 [.36, 1.45] |

*Note. * indicates statistical significance. CI not containing 0 indicates significant coefficient. CI not containing 1 indicates significant risk ratio. Smoking status, body mass index, depression, age, sex, race/ethnicity, education, marital status, and work status were included as covariates. Good sleepers were used as the reference group. Standardized coefficients are presented.*

**Supplemental Table 4.** Log-binomial regression of categorical sleep dimensions predicting cardiovascular conditions in M2 and MR samples.

|  | **M2** | | **MR** | |
| --- | --- | --- | --- | --- |
| **Categorical sleep dimensions** | **β [CI]** | **Risk Ratio [CI]** | **β [CI]** | **Risk Ratio [CI]** |
| Irregular (reference group: regular) | -.05 [-.22, 12] | .95 [.90, 1.12] | .04 [-.29, .37] | 1.04 [.75, 1.45] |
| Dissatisfied (reference group: satisfied) | .34* [.21, .48] | 1.41* [1.22, 1.62] | .32* [.03, .61] | 1.38* [1.03, 1.84] |
| Frequent naps (reference group: infrequent naps) | .25* [.13, .37] | 1.28* [1.14, 1.44] | -.07 [-.32, .18] | .93 [.72, 1.19] |
| Inefficient (reference group: efficient) | .24* [.11, .37] | 1.27* [1.12, 1.44] | .39* [.11, .67] | 1.48* [1.11, 1.96] |
| Suboptimal duration (reference group: appropriate duration) | .26* [.13, .40] | 1.30* [1.14, 1.49] | .27 [-.05, .59] | 1.31 [.95, 1.80] |

*Note.* * indicates statistical significance based on raw CI. CIs not containing 0 indicates significant coefficient. CI not containing 1 indicates significant risk ratio. Smoking status, body mass index, depression, age, sex, race/ethnicity, education, marital status, and work status were included as covariates.

**Supplemental Table 5.** Incremental prediction of cardiovascular conditions incidence by latent sleep classes over individual sleep characteristics.

|  | **M2 sample** | | | **MR sample** | | |
| --- | --- | --- | --- | --- | --- | --- |
| **Step 1. Individual sleep characteristics** | B | S.E. | p | B | S.E. | p |
| Model 1: Irregularity (vs. regularity) | -0.02 | 0.11 | 0.89 | 0.06 | 0.17 | 0.73 |
| Model 2: Dissatisfaction (vs. satisfaction) | **0.45** | **.10** | **<.001** | **.40** | **0.15** | **0.009** |
| Model 3: Frequent naps (vs. infrequent naps) | **0.37** | **0.08** | **<.001** | -0.07 | 0.13 | .60 |
| Model 4: Inefficiency (vs. efficiency) | **0.35** | **.10** | **<.001** | **0.33** | **0.15** | **0.03** |
| Model 5: Poor duration (vs. appropriate duration) | **0.42** | **0.11** | **<.001** | 0.19 | 0.17 | 0.27 |
| **Step 2. Sleep class membership added** | B | S.E. | p | B | S.E. | p |
| **Sleep classes in Model 1 (irregularity)** |  |  |  |  |  |  |
| Dissatisfied/inefficient (vs. good sleeper) | **0.36** | **0.12** | **0.003** | **0.63** | **0.13** | **<.001** |
| Napper (vs. good sleeper) | **0.88** | **0.12** | **<.00** | **0.45** | **0.14** | **0.001** |
| Irregular (vs. good sleeper) | **0.47** | **0.23** | **0.04** | -0.03 | 0.33 | 0.93 |
| **Sleep classes in Model 2 (dissatisfaction)** |  |  |  |  |  |  |
| Dissatisfied/inefficient (vs. good sleeper) | 0.20 | 0.12 | 0.09 | **0.64** | **0.13** | **<.001** |
| Napper (vs. good sleeper) | **0.73** | **0.11** | **<.001** | **0.47** | **0.14** | **0.001** |
| Irregular (vs. good sleeper) | **-0.31** | **0.12** | **0.009** | -0.22 | 0.32 | 0.49 |
| **Sleep classes in Model 3 (nap frequency)** |  |  |  |  |  |  |
| Dissatisfied/inefficient (vs. good sleeper) | 0.21 | 0.12 | 0.07 | **0.65** | **0.13** | **<.001** |
| Napper (vs. good sleeper) | **0.68** | **0.11** | **<.001** | **0.50** | **0.14** | **<.001** |
| Irregular (vs. good sleeper) | **-0.31** | **0.12** | **0.01** | -0.26 | 0.33 | 0.44 |
| **Sleep classes in Model 4 (inefficiency)** |  |  |  |  |  |  |
| Dissatisfied/inefficient (vs. good sleeper) | 0.21 | 0.12 | 0.08 | **0.66** | **0.13** | **<.001** |
| Napper (vs. good sleeper) | **0.73** | **0.11** | **<.001** | **0.47** | **0.14** | **0.001** |
| Irregular (vs. good sleeper) | **-0.31** | **0.12** | **0.01** | -0.21 | 0.32 | 0.51 |
| **Sleep classes in Model 5 (duration)** |  |  |  |  |  |  |
| Dissatisfied/inefficient (vs. good sleeper) | 0.21 | 0.12 | 0.08 | **0.63** | **0.13** | **<.001** |
| Napper (vs. good sleeper) | **0.72** | **0.11** | **<.001** | **0.47** | **0.14** | **0.001** |
| Irregular (vs. good sleeper) | **-0.31** | **0.12** | **0.01** | -0.21 | 0.32 | 0.51 |

*Note.* Separate models were run for each sleep dimension as a predictor of cardiovascular conditions incidence above and beyond the sociodemographic and risk factor covariates (step 1). Good sleepers were used as a comparison group for the other three profiles in predicting outcomes, above and beyond individual sleep dimensions (step 2). Bold text indicates significance (p<.05).
